# Methyl coenzyme M reductase as a target for inhibition of methanogenesis in ruminants: challenges and opportunities

**DOI:** 10.3389/fmicb.2025.1704809

**Published:** 2026-01-29

**Authors:** Zhiyong Shao, Zheng Lu, Lijun Wu, Wei Liu, Muhammad Abdullah, Faiz-ul Hassan, Xiabing Chen, Ruige Cao, Bin He

**Affiliations:** 1 Institute of Animal Husbandry and Veterinary, Wuhan Academy of Agricultural Sciences, Wuhan, Hubei, China; 2 Faculty of Animal Science and Technology, Yunnan Agricultural University, Kunming, China; 3 College of Veterinary Medicine, Northwest A&F University, Yangling, Shaanxi, China; 4 Department of Veterinary Pharmacology, College of Veterinary Medicine, Huazhong Agricultural University, Wuhan, China; 5 Department of Microbiology, Cholistan University of Veterinary & Animal Sciences, Bahawalpur, Pakistan; 6 Department of Breeding and Genetics, Cholistan University of Veterinary & Animal Sciences, Bahawalpur, Pakistan

**Keywords:** 3-nitrooxypropanol, bromoethanesulfonate, greenhouse gasses, methanogenesis, methyl-coenzyme M reductase, phytochemicals, post-translational modifications

## Abstract

Enteric methane from ruminants is a major source of greenhouse gasses (GHG) emissions globally, and its formation also leads to a decrease in animals’ productivity due to loss of dietary energy. Reducing enteric methane emissions is essential for mitigating greenhouse gas–driven climate changes while simultaneously enhancing ruminant production efficiency. Methanogens residing in the rumen are responsible for enteric methane production. They reduce carbon dioxide to methane with the help of hydrogen, thus playing a crucial role in global methane emissions. Methyl coenzyme M reductase (MCR) is a key enzyme in methanogens that catalyzes the final step of methanogenesis. This review consolidates information on MCR enzyme’s structure, cofactor chemistry, and post-translational modifications (PTMs), followed by a critical appraisal of inhibition strategies using synthetic compounds like 3-nitrooxypropanol (3-NOP) and bromoethanesulfonate (BES) along with their mode of action. Modern *in silico* studies for the identification of novel natural MCR inhibitors have also been discussed. Blocking MCR through synthetic or natural compounds is a promising approach for mitigating methane emissions from ruminants, allowing the rest of the rumen’s microbial community to function normally. By specifically blocking MCR, hydrogen and other byproducts of carbohydrate fermentation are still consumed, allowing the animal’s digestion and productivity to remain unaffected while significantly reducing its contribution to greenhouse gas emissions. Making it a target, the issue of methane emission in ruminants can be solved without affecting the overall rumen microbiota. Moreover, challenges (hydrogen accumulation, cost, and regulatory hurdles) and emerging opportunities regarding MCR inhibitory strategies are proposed to guide targeted research for scalable methane mitigation in ruminants.

## 1 Introduction

The enteric fermentation occurs due to the activity of microbial communities that reside inside the ruminant’s rumen (Montoya-Flores et al., 2020). The collective action of these microorganisms facilitates the conversion of complex macromolecules present in feed into smaller molecules, including volatile fatty acids (VFAs) (Williams et al., 2019). These VFAs represent the primary source of energy for ruminants (Wang et al., 2020). In addition to VFAs, other products such as carbon dioxide (CO_2_) and hydrogen (H_2_) are also produced (Niwińska, 2012). In the rumen, a specialized group of microorganisms known as methanogens is found, which consume this H_2_ and CO_2_ to produce methane (CH_4_) (Sirohi et al., 2010). This process of converting H_2_ and CO_2_ into methane is known as methanogenesis (Lyu et al., 2018). However, the methane produced as a result is a potent greenhouse gas and a leading source of global warming (Van Amstel, 2012; Zhang et al., 2024). Over 100 years, methane has been considered 28 times more effective at trapping heat in the atmosphere than CO_2_ (Mohajan, 2011).

The enzyme responsible for producing this methane by methanogens is methyl-coenzyme M reductase (MCR) (Ermler et al., 1997; Dinh and Allen, 2024). Some compounds have been developed to inhibit methane production by specifically targeting MCR (Martínez-Fernández et al., 2014; Hassan et al., 2023). In ruminants, the most successful and well-known inhibitor to date is 3-nitrooxypropanol (3-NOP) (Orzuna-Orzuna et al., 2024). Various *in vitro* trials have proved that 3-NOP decreases methane production to a significant level (Alvarez-Hess et al., 2019; Romero-Pérez et al., 2016). In dairy cows, dietary supplementation with it was also found to be effective for reducing methane yields (Kebreab et al., 2023). This represents a promising approach for mitigating methane emissions from ruminants, allowing the rest of the rumen’s microbial community to function normally (Ginovska et al., 2025; Choudhury et al., 2015). By specifically blocking MCR, hydrogen and other byproducts of carbohydrate fermentation are still consumed, allowing the animal’s digestion and productivity to remain unimpaired while significantly reducing its contribution to greenhouse gas emissions (Hassan et al., 2021; Marmier and Schosger, 2020; Su et al., 2023). There is still a big concern about how 3-NOP affects the growth performance, ruminal pH, and methanogenesis at lower concentrations, as many studies used it as a feed additive at higher dosages (100 to 200 mg/kg DM) just for methane inhibition (Vyas et al., 2016; Alemu et al., 2023). Most of the trials have been conducted only on dairy cows, and very limited data are available on beef cattle, sheep, or goats (Dijkstra et al., 2018; Kim et al., 2020).

Although several synthetic compounds have been tested against purified MCR to inhibit its activity, no significant efforts have been made to test them on animal models (except 3-NOP). Testing compounds against purified MCR is a big challenge, as it gets inactivated in the presence of oxygen (Singh et al., 2003). Moreover, there is a lack of literature about the precise mechanism by which post-translational modifications (PTMs) in MCR affect interactions with the known inhibitors. This review aims to summarize current knowledge on MCR structure and function along with its inhibition with known inhibitors. Recent studies that involve modern *in silico* approaches to discover potent natural inhibitors against MCR are also summarized. The major aim of this comprehensive review is to provide practical insights about structural features of the MCR enzyme and to design inhibitory strategies using synthetic and natural compounds to block its active site, subsequently leading to blocking/mediating the methanogenesis pathway.

## 2 Methane emission from ruminants

According to the Food and Agriculture Organization of the United Nations (FAO), there were approximately 1.6 billion cattle, 1.23–1.26 billion sheep, and 1–1.1 billion goats worldwide in 2023. FAO has also reported that an average of about 600 million tons of CH_4_ gas is emitted into the environment every year, and a major portion of it originates from the agricultural sector (FAO, 2023). According to the U. S. Environmental Protection Agency (EPA) report in 2022, almost 25% of anthropogenic methane emissions are contributed by the livestock sector. With the help of the Tier 2 method, it was estimated that global methane emissions from the livestock sector had increased from 31.8 Tg yr.^−1^ in 1890 to 131 Tg yr.^−1^ in 2019. This increase is directly related to the expansion of the livestock population to meet the rising demand for milk and meat products (Zhang et al., 2022). In 2019, the livestock sector of China emitted approximately 37.5–61.7 Tg of methane (Du et al., 2024). In India, methane emissions from the livestock sector in 2019 were estimated at 11.63 Tg (Samal et al., 2024). From 2010–2017, the livestock sector of North America produced an average of 36.9 Tg of methane per year (Lu et al., 2022). Another drawback of enteric methane production is that it has a negative impact on animal productivity, resulting in a loss of total gross energy intake ranging from 2 to 12% (Ebeid et al., 2020; Tangjitwattanachai et al., 2015). These numbers suggest that the livestock sector shares a major portion of methane gas into the environment, and there is an urgent need to develop effective mitigation strategies.

## 3 Hydrogenotrophic methanogenesis

**Figure 1:**
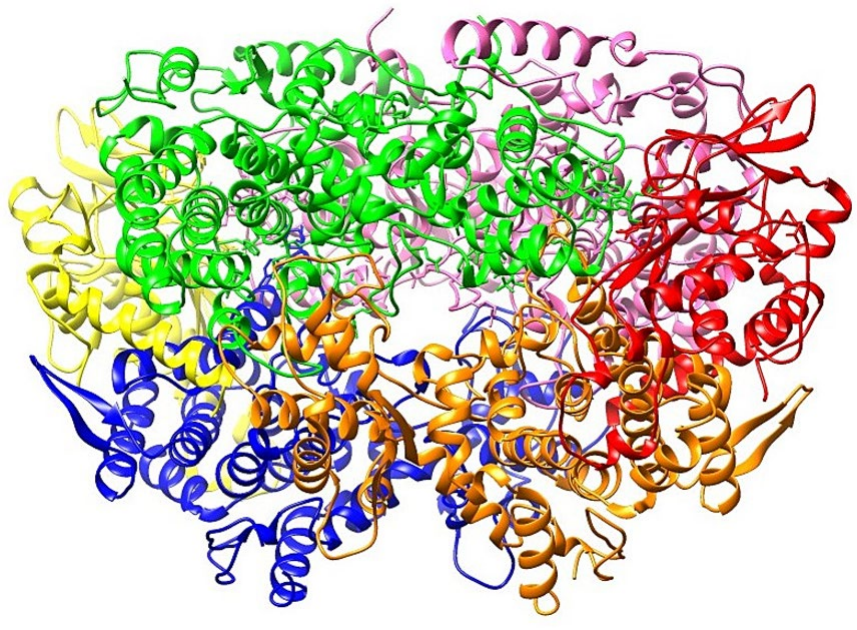
Functional role of MtrA-H complex and MCR in the Wolfe pathway (Thauer, 2012).

Rumen methanogens produce methane through hydrogenotrophic methanogenesis. Two scientists, Wolfe and Rouviere, were the first to study this methane-forming process in microorganisms that utilize H_2_ and CO_2_. They proposed that during the complete metabolic cycle (Figure 1), the energy remains conserved (Ragsdale and Pierce, 2008). The coenzymes found solely in methanogens are used as C1-carriers, including methanofuran, tetrahydromethanopterin (H4MPT), and coenzyme M (CoM-SH) (Thauer, 2012). Ferredoxin is an essential electron carrier that reduces CO_2_ to formyl methanofuran along with the inflow of Na^+^ ions at earlier stages. A V-type ATP synthase induces the generation of ATP with this Na^+^ gradient. However, the reduction of this oxidized ferredoxin with H_2_ is thermodynamically unfavorable due to the greater redox potential compared to H_2._ That is why methanogens use energy coupling (electron bifurcation and ion gradient) to reduce ferredoxin (Thauer, 2010). For the endergonic reduction of oxidized ferredoxin, a total of three exergonic reactions occur during the whole Wolfe pathway (Thauer et al., 2008). In the first exergonic reaction, tetrahydro methano protein transfers its methyl group to coenzyme M (CoM-SH) with the help of H4MPT methyl-transferase (MtrA-H) complex, leading to the formation of methyl coenzyme M (CH_3_-S-CoM). This step is coupled with the translocation of Na^+^ ions that drive ATP synthesis (Gottschalk and Thauer, 2001). In the second exergonic reaction, methyl coenzyme M is reduced by methyl coenzyme M reductase (MCR) using coenzyme B (CoB-SH), releasing a methane molecule (Scheller et al., 2010). A heterodisulfide is also formed that is recycled back to coenzyme M and coenzyme B using the cytosolic hydrogenase heterodisulfide reductase complex in the third exergonic reaction, which involves H_2_ to drive endergonic reduction of ferredoxin and exergonic reduction of CoM-S-S-CoB (Grüber et al., 2014).

## 4 Identified species of rumen methanogens

Since the discovery of methanogenic archaea in the rumen, only a few species of ruminal methanogens have been isolated into pure cultures (Jarvis et al., 2000). These include *Methanobacterium formicicum*, *Methanobacterium bryantii*, *Methanobrevibacter ruminantium*, *Methanobrevibacter millerae*, *Methanobrevibacter olleyae*, *Methanomicrobium mobile*, *Methanoculleus olentangyi*, and *Methanosarcina barkeri* (Janssen and Kirs, 2008; Sirohi et al., 2010). Two new species of ruminal methanogens have also been isolated in recent years. These include *Methanobrevibacter boviskoreani*, which was isolated from the rumen of Korean native cattle (Lee et al., 2013) and *Methanoculleus bourgensis*, which was isolated from the rumen of Holstein cattle (Battumur et al., 2019). It has been reported that 92.3% of these methanogens residing in the rumen belong to three major genera, as determined by 16S rRNA gene sequencing for identification and quantification of rumen methanogens (Janssen and Kirs, 2008). *Methanobrevibacter* is the largest group accounting for 61.6% of methanogens, while *Methanomicrobium* and an unidentified genus account for 14.9 and 15.8%, respectively (Table 1). At the species level, *Methanobrevibacter gottschalkii*, *Methanobrevibacter thaueri*, and *M. millerae* occupy the largest portion of the *Methanobrevibacter* genus (33.6%), while *M. ruminantium* and *M. olleyae* account for 27.3% of this genus (Janssen and Kirs, 2008). Duin et al. (2016) studied the effect of 3-NOP on pure rumen methanogen cultures and reported that different dosages are required for different species to completely inhibit their growth and methane production. The effective dose against *Methanobrevibacter ruminantium* was less than 0.25 μM. For *Methanomicrobium mobile* and *Methanosarcina barkeri,* the dose was less than 10 μM. For *Methanobrevibacter millerae*, the effective dose was 1 μM. This indicates that 3-NOP not only inhibits MCR but also influences the growth of methanogens. Romero et al. (2023) reported that the effect of bromoform, a natural compound extracted from *Asparagopsis taxiformis,* on pure rumen methanogens was that a dose of 2–10 μM inhibited the growth of *M. smithii, M. ruminantium, M. stadtmanae, M. barkeri, M. millerae, M. wolfei*, and *M. mobile*. Although bromoform inhibits the growth of rumen methanogens, its effect on MCR is still unknown.

**Table 1:** Representing different rumen methanogen genera along with their relative abundance in the rumen and MCR isoenzymes present in each methanogenic species.

Genus	Percentage	Identified Species	Isoenzyme (MCR I or II)	References
*Methanobacterium*	1–2%	*M. bryantii*	I & II	Janssen and Kirs (2008) and Sirohi et al. (2010)
*Methanobrevibacter*	61.6%	*M. ruminantium*	I	Khairunisa et al. (2023), Janssen and Kirs (2008), and Attwood et al. (2020)
		*M. olleyae*	I & II	
		*M. thaueri*	I	
		*M. millerae*	I & II	
		*M. bovikoreani*	I	
		*M. gottschalkii*	I & II	
		*M. wolinii*	I	
		*M. smithii*	I & II	
		*M. woesei*	I	
*Methanosarcina*	1–2%	*M. barkeri*	I	Lambie et al. (2015) and Jeyanathan et al. (2011)
*Methanomicrobium*	14.9%	*M. mobile*	I	Janssen and Kirs (2008) and Khairunisa et al. (2023)

## 5 Structural and mechanistic insights into MCR

### 5.1 Protein architecture and encoding genes

**Figure 2:**
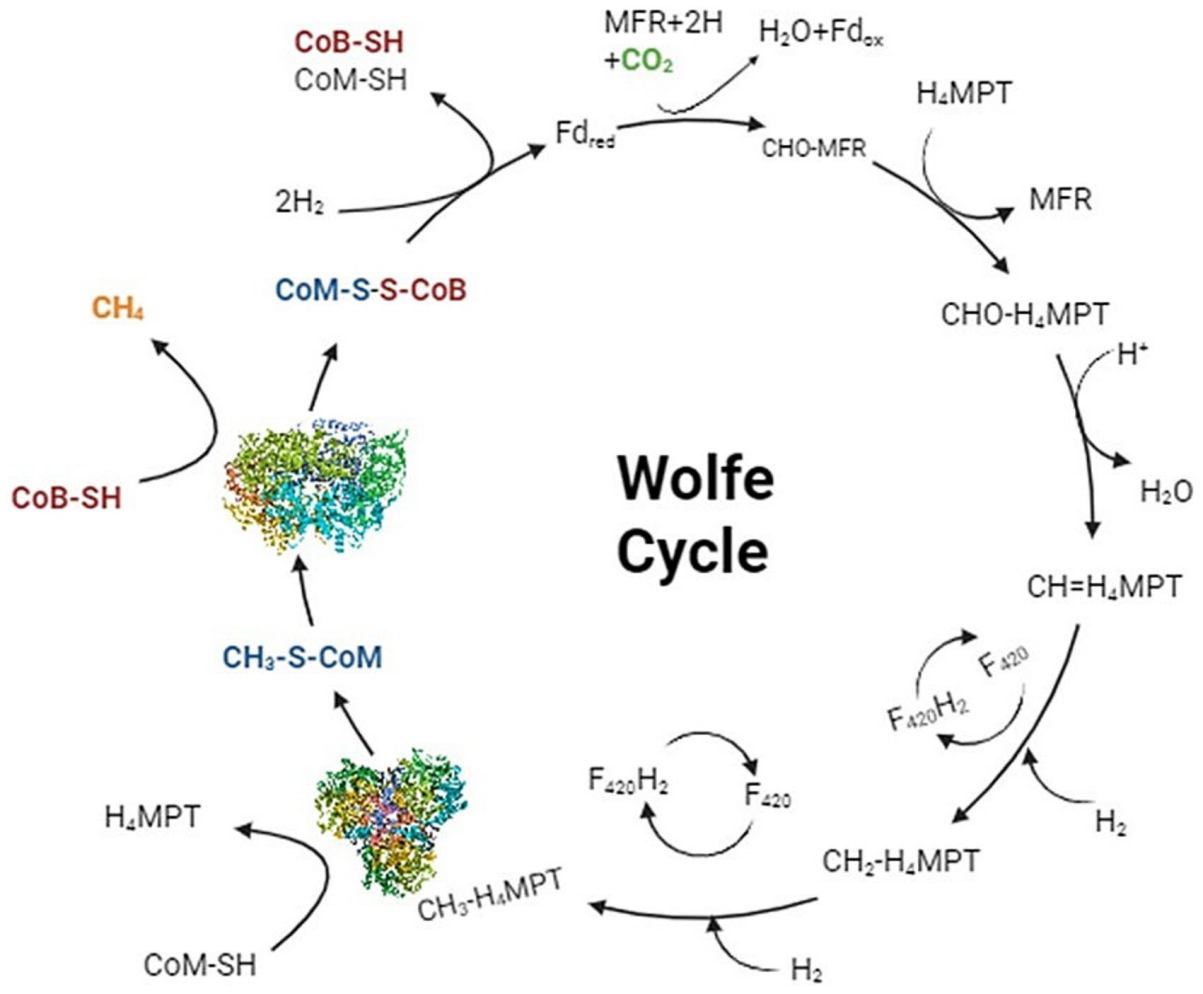
Six chains of MCR, including *α* (green), α′ (pink), *β* (blue), β′ (orange), *γ* (yellow), and γ′ (red), form a hetero-hexamer (Ermler et al., 1997).

The molecular mass of MCR is 300 kD, consisting of three subunits organized in an *α_2_ β_2_ γ_2_* configuration (Figure 2), forming a hetero-hexamer (Ermler, 2005).

The active sites of MCR are two deep channels with a depth of 30 Å. Both active sites are hydrophobic in nature and are separated by a distance of 50 Å (Figure 2) (Ginovska et al., 2025). Within each active site, an independent tetrapyrrole molecule known as coenzyme F_430,_ with a molecular mass of 905 Da, is buried (Figure 2; Ermler et al., 1997). LeGall was the first one to observe the coenzyme F_430_ as yellow chromatogram bands from cell extracts (Wolfe, 1985). Later on, Ellefson et al. (1982) described its presence in MCR. In the center of this molecule, a nickel hydrocorphinoid cofactor is present (Harmer et al., 2008; Selmer et al., 2000; Diekert et al., 1980). The presence of nickel ions in the molecule’s structure is essential for the proper functioning of MCR (Ebner et al., 2010). During methanogenesis, the nickel center of F_430_ undergoes a redox change, specifically transitioning from Ni(I) to Ni(III) (Prakash et al., 2014; Laird et al., 2024), facilitating the cleavage of the methyl-thioether bond to release the methyl group from methyl coenzyme M and reducing it with an electron from coenzyme B for methane formation (Ebner et al., 2010).

At the genetic level, operon *mcrBDCGA* encodes for the MCRI isoenzyme, whereas operon *mrtBGDA* encodes for the MCRII isoenzyme. This gene order is highly conserved over decades in methanogenic archaea (Thauer, 2019), and the MCR is assembled in strict accordance with this arrangement (Hallam et al., 2003; Klein et al., 1988). According to this, McrB is the first to be translated, followed by McrD (Klein et al., 1988). Both complexes get merged with McrG and McrA after their translation. Due to post-translational modification and attachment with coenzyme F_430_, the McrD unit is weakened or lost in the process (Lyu et al., 2018). Further studies are required to explore the specific functions of each unit and how they interact (Wagner et al., 2017). The expression of both isoenzymes is regulated by the availability of H_2_ and CO_2_. Under normal or elevated H_2_/CO_2_ conditions, methanogens predominantly express MCRII, which exhibits higher catalytic function (Enoki et al., 2011). When ratio of H_2_/CO_2_ concentration decreases, methanogens shift to MCRI, which functions efficiently even under depleted conditions, because methanogens face an energy deficit and cannot support the energetically more expensive form of enzyme, i.e., MCRII. These shifts during high and low availability of gasses allow methanogens to survive (Bonacker et al., 1993).

### 5.2 Different EPR states of MCR

**Figure 3:**
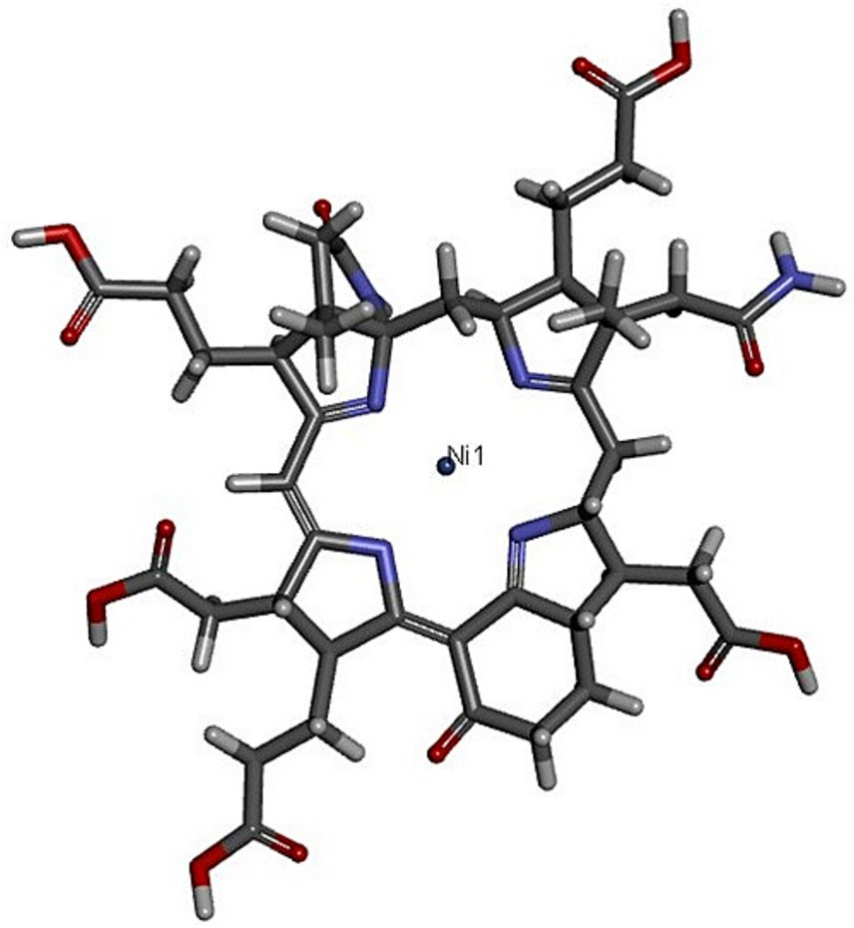
Three-dimensional structure of coenzyme F_430,_ having a Ni at the center (Grabarse et al., 2000).

Several studies have revealed that the nickel hydrocorphinoid cofactor (Ni) (Figure 3) is essential for final methane forming reaction mediated by MCR. As this Ni exists in several different oxidation states, the electron paramagnetic resonance (EPR) studies are quite helpful for the researchers to study the MCR’s redox chemistry and catalytic mechanism (Ebner et al., 2010). Over the last four decades, researchers have revealed several EPR detectable and EPR silent states, indicating a specific oxidation or coordination state of nickel. In the case of MCR, these states were first revealed by Simon Albracht while studying *Methanothermobacter marburgensis* (Albracht et al., 1988). Under growth conditions of 65 °C and an atmosphere containing 80% H_2_/20% CO_2_, *M. marburgensis* exhibits an exponential growth with a doubling time of 2 h and methane formation at a rate of 10 μmol min^−1^ mg protein^−1^. At this point, the cell extracts are considered to be fully active and are making methane at a rate of 10 μmol min^−1^ mg protein^−1^. Earlier, when the enzyme was extracted from grown cells, it only showed an activity of 0.1 μmol min^−1^ mg protein^−1^. Later on, it was observed that when the cell suspension of *M. marburgensis* was gassed with 100% H_2,_ its extract exhibited a strong MCR-red1 signal, with methane production at a rate of 2 μmol min^−1^ mg (Albracht et al., 1988) confirming the fact that for enzyme’s activity, it should be in MCR-red1 state (Rospert et al., 1991). After 10-fold purification of MCR from the cell extract and in the presence of the competitive inhibitor coenzyme, it catalyzed methane formation using methyl-coenzyme M and coenzyme B at a rate of about 20 μmol mn^−1^ mg protein^−1^. This purified MCR had a greenish color with a light absorption peak at 386 nm (in UV–visible spectrum) and exhibited the axial MRCred1 signal having a spin concentration near 0.2. These MCR-red1 values were in excellent agreement with those generated by using a synthetic penta-methyl ester of Ni(I) F_430_. Therefore, according to these observations, it was concluded that nickel in MCR-red1 exists in the +1 oxidation state (Jaun and Pfaltz, 1986). When the enzyme was inactivated with the help of chloroform, the spin concentration and the specific activity decreased in parallel. This further proved that Ni in MCR should be in +1 oxidation state (Ni(I)) for catalytic activity (Rospert et al., 1991).

When *M. marburgensis* was grown on 80% H_2_/20% CO_2_ and the culture was gassed with CO_2_ right before MCR extraction, a strong MCR-ox1 EPR signal was observed by the enzyme, but it showed no activity (Mahlert et al., 2002; Goubeaud et al., 1997). Alternatively, this MCR-ox1 signal can also be induced by treating the culture with 20 mmol sodium sulfide rather than gassing it with 80% N_2_/20% CO_2_. This approach has been demonstrated by using *M. marburgensis* and *Methanosarcina thermophila* cultures (Becker and Ragsdale, 1998). The ox1 EPR signal generated by inactive MCR was identical to red1 EPR signal of active MCR but stability of MCR in ox1 is far greater compared to MCR-red1 state. This signal can further be extinguished with the help of oxygen and other compounds (chloroform and nitric oxide), but it is needed at much higher concentrations (Singh et al., 2003). This stability helps researchers to extract the enzyme safely from the cells. Researchers observed that when the MCR in the ox1 state in the presence of methyl-coenzyme M and titanium (III) citrate along with a pH 9, it can be converted to active MCR-red1. The experiment using Ti(III) to convert MCR-ox1 to MCR-red1 was done to indicate that in MCR-ox1, the nickel is in the 3 + oxidation state, i.e., Ni(III) (Goubeaud et al., 1997).

In addition to these states, two more states designated as ox2 and ox3 have also been observed. When the MCR-red1 state is treated with Na_2_SO_3_, it gives a light sensitive EPR signal designated as MCR-ox2 and when MCR-red1 is treated with O_2_ it gives a light sensitive signal known as MCR-ox3. These two states are irreversible as MCR-red1 state still cannot be achievable after cutting down the supply of O_2_ and Na_2_SO_3._ In these states, the Ni is also in the +3 oxidation state, as observed in the MCR-ox1 state (Mahlert et al., 2002).

### 5.3 Mechanism of action of MCR

**Figure 4:**
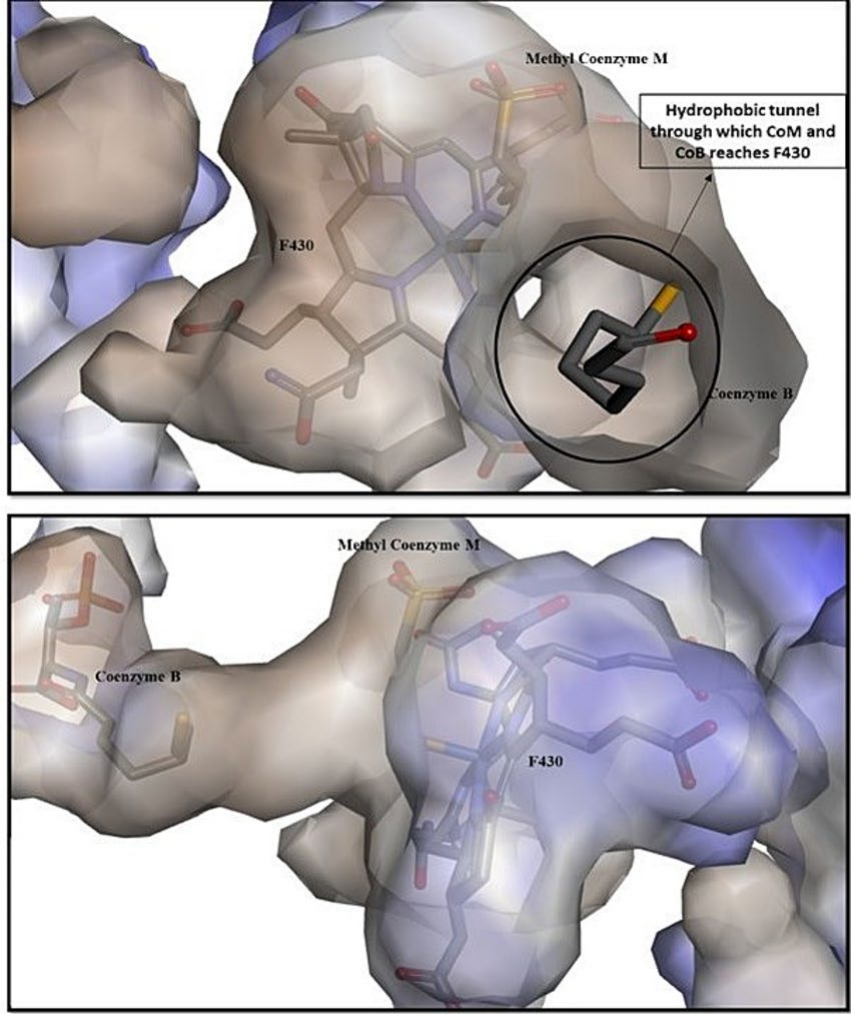
Entry and binding of methyl coenzyme M and coenzyme B within the hydrophobic active site of MCR. The position of methyl coenzyme M closer to the coenzyme F_430_ gives evidence that it enters first within the hydrophobic cavity compared to coenzyme B (Goenrich et al., 2004).

The x-ray studies of MCR’s active site revealed that both of the substrates, methyl coenzyme M (CH_3_-S-CoM) and coenzyme B (CoB or HS-CoB), enter through the same narrow channel, which opens into the hydrophobic cavity above the hydrocorphinoid plane of F_430_ (Figure 4; Cedervall et al., 2011; Grabarse et al., 2000). It is the phosphate group of CoB which makes the ionic interactions with the residues of MCR and stabilizes itself within the binding pocket while its thiol group is positioned around 8.7 Å from the nickel. The CH_3_-S-CoM, with the methyl group, is found more deeply within the channel, suggesting that it may enter the channel first than CoB for efficient chemical reaction (Ebner et al., 2010). After binding with the key residues of MCR, CoB undergoes a series of conformational change that brings the methyl coenzyme M into closer proximity to the nickel, and this promotes C-S bond cleavage.

**Figure 5:**
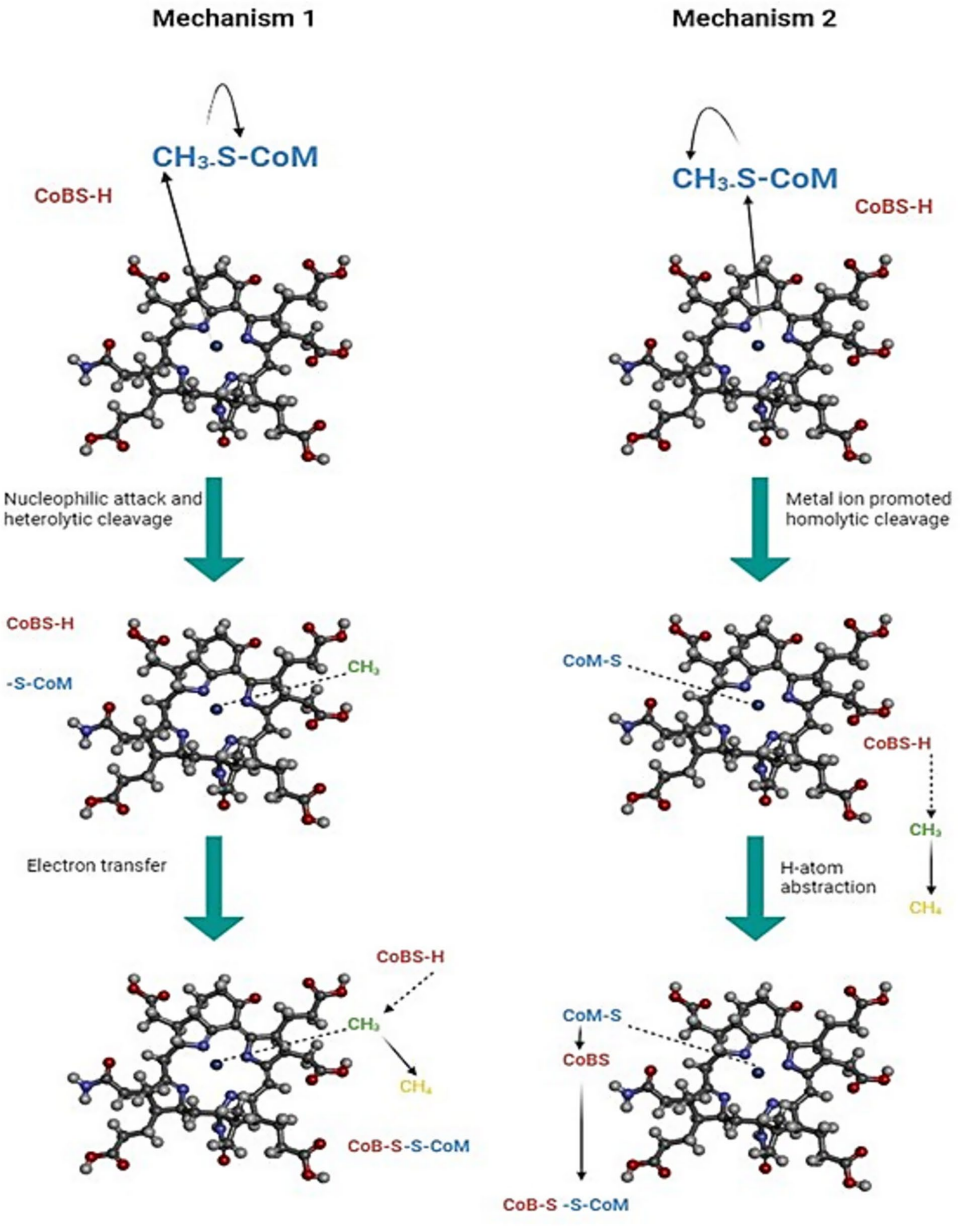
Two proposed catalytic mechanisms of methyl-coenzyme M reductase (MCR). In mechanism 1, Ni(I) forms a transient Ni–methyl organometallic intermediate by binding the methyl group from methyl coenzyme M (CH_3_–S–CoM). Hydrogen atom transfer from coenzyme B (HS–CoB) then releases CH_4_, and coenzyme M and coenzyme B (CoM & CoB) form the heterodisulfide (CoM–S–S–CoB). In mechanism 2, Ni(I) induces homolytic cleavage of methyl coenzyme M (CH_3_–S–CoM), generating a methyl radical (CH_3_). This radical abstracts a hydrogen atom from coenzyme B (HS–CoB) to form CH_4_, while the remaining thiyl species combine to form CoM–S–S–CoB (Ermler, 2005).

Different transient kinetic experiments revealed that MCR indeed can bind either substrate independently; however, only one binary complex, MCR and CH_3_-S-CoM, is productive, whereas with the other MCR and HS-CoB, it is inhibitory (Jablonski et al., 1990). The main reason for it is that HS-CoB (coenzyme B) blocks the active site of MCR, inhibiting the entry of bulk solvent (Wongnate and Ragsdale, 2015). The role of nickel in the MCR catalytic cycle is still not fully understood, and two competing catalytic mechanisms for MCR are in debate (Figure 5; Yang et al., 2007). In mechanism I, after the binding of CH_3_-S-CoM and HS-CoB within the active site of MCR, Ni(I) of F_430_ performs a nucleophilic attack on the methyl group of CH_3_-S-CoM, leading to the formation of a methyl-Ni(III) intermediate. The HS-CoM donates its single electron to this intermediate, resulting in a methane molecule. A heterodisulfide bond is formed between the two coenzymes, which are then recycled back (Signor et al., 2000). This mechanism is theorized based on mechanistic work with the F_430_ model complexes (Lahiri and Stolzenberg, 1993), location of both substrates within the active site of inactive Ni(II) MCR (Ermler et al., 1997), and crystallographic studies of the active Ni(I) enzyme with 3-bromopropanesulfonate and methyl halide (Yang et al., 2007). In mechanism II, the sulfur atom of CH_3_- S-CoM gets attacked by Ni(I) resulting a homolytic cleavage of the methyl-sulfur bond, generating a free methyl radical and a Ni(II)-thiolate complex. The HS-CoB as in mechanism I, transfers its electron to this methyl radical leading to the formation of a methane molecule. This mechanism is based on density functional theory calculations (Chen et al., 2012) and studies of the isotope effects of the MCR reaction with CH_3_-S-CoM and homologous substrate C_2_H_5_-S-CoM (Scheller et al., 2013).

## 6 Functional role of post-translational modifications in MCR

Post-transitional modifications (PTMs) in MCR have been known for over 20 years, and they represent one of the most significant characteristics of this enzyme (Kahnt et al., 2007). These were first reported in 1997 during studies of MCRI from *Methanobacterium thermoautotrophicum* (Ermler et al., 1997). PTMs observed in the methanogenic MCR are 1-N-methylhistidine, S-methylcysteine, 2-(S)-methylglutamine, 5-(S)-methylarginine, thioglycine, and didehydroaspartate (Chen et al., 2020).

Earlier it was believed that methyl group from coenzyme M is responsible for the methylation of these amino acids but later on, Selmer et al. (2000) ruled out this possibility, and proved that methyl group of S-adenosylmethionine (SAM) was the main agent behind these methylations. They proved this fact by growing *M. thermoautotrophicum* in the media with l-(methyl-D3) methionine. This study also declared that SAM was not used as the carbon source for methanogenesis and could not be converted into methane. Recently, a gene (mcmA) that codes for SAM-dependent methyltransferase has been identified for cysteine methylation, and it has been proposed that cysteine methylation helps to survive mesophilic conditions (Nayak et al., 2020). There are limitations regarding methylation done by the SAM mechanism, as it could not explain the methylation of 2-(S)-methylglutamine and 5-(S)-methylarginine (Liscombe et al., 2012).

Among all other PTMs, arginine methylation has been observed across all methanogenic archaea (Wagner et al., 2016; Wagner et al., 2017). This PTM was studied in *Methanococcus maripaludis* mutant strains in which mmpX gene was removed which codes for Mmp10 protein. This protein is responsible for arginine methylation in MCR. The resulting MCR enzyme lacked arginine methylation and was thermally unstable in cell extracts, with a loss of almost 90% of activity within 1 h (Lyu et al., 2020). The assumed function of 3-methylhistidine is that it may help in better positioning of the histidine imidazole ring that suits CoB. The pKa of histidine is also altered by this methylation, thereby helping CoB to bind more tightly within the active site (Grabarse et al., 2000).

A study related to didehydroaspartate have shown that the carboxyl group of this modified residue interacts with His_484_ and Trp_427_ which helps in stabilizing the loop regions to which CoM and CoB binds. The addition of a double bond to an aspartate has an influence on the active site as well as the catalytic activity of MCR. The Cα-Cβ double bond narrows the conformational space of the side chain, fixing the carboxylate group in a single direction and reducing its mobility, as well as lowering the pKa value (Wagner et al., 2016).

Thioamidation has been observed in both methanogenic and methanotrophic MCRs. Earlier two proteins YcaO and TfuA have been hypothesized for the thioamidation of glycine. Recent roles of these two proteins have been studied using genetic and mass spectrometry approaches, which revealed that they both are responsible for glycine thioamidation. Removal of both genes caused the absence of thioamidation of Gly_465_ at the active site of MCR (Nayak et al., 2017). There are different theories regarding the function of thioamidation: (1) it helps in catalysis by acting as an intermediate electron carrier (Horng et al., 2001); (2) it helps in the reduction of the pKa value for the sulfhydryl group to assist the deprotonation of CoB (Grabarse et al., 2000); and (3) it enhances the enzyme stability (Nayak et al., 2017).

## 7 MCR inhibition

Some compounds have been developed to inhibit methane production in ruminants by targeting MCR, either through synthetic compounds or using natural compounds, mainly phytochemicals.

### 7.1 Synthetic inhibitors

A few inhibitors have been synthesized to suppress MCR’s activity by oxidizing the nickel present at the center of the active site (Table 2). Among these, 3-nitroxypropanol and bromoethanesulfonate (BES) are the most studied and extensively tested to inhibit methane production. 3-NOP is a synthetic compound that has been studied in the livestock sector without any known adverse effects on animal health (Martínez-Fernández et al., 2014). It inhibits the activity of MCR to suppress methane emissions from the agricultural sector. 3-NOP acts as a structural analog of CH_3_-S-CoM in size and polarity. After entering the methanogenic cell, it binds with the hydrophobic active site of MCR and arranges its reducible nitrate group at an electron transfer distance to Ni(I). The Ni(I) donates its electron to this nitrooxy group and becomes oxidized, i.e., Ni(III). After receiving the free electron from Ni, 3-NOP gets reduced to nitrite (Duin et al., 2016). Studies in both dairy and beef cattle have consistently demonstrated methane reductions averaging 30–40%, with some trials reporting decreases of up to 60%, depending on dose, diet, and experimental conditions (Reynolds et al., 2014; Kebreab et al., 2023). Several other nitro compounds also showed promising results in *in vitro* rumen fermentation (Table 2), and it is believed that the compounds having a nitro group can strongly oxidize the Ni in MCR. Saro et al. (2025) reported the effect of 3-NOP on lactating cows, showing that its use in the feed mitigates methane production to some extent. Lahart et al. (2025) reported decreased methane production and increased H_2_ in cows treated with 3-NOP. Almeida et al. (2023) observed that using 3-NOP in the feed of cattle increased total VFA concentration. This proves the fact that 3-NOP not only decreases methane production but increases H_2_ and VFA production as well.

**Table 2:** Major synthetic compounds with the potential of decreasing CH_4_ production by targeting purified MCR enzyme or by decreasing methanogenic populations in *in vitro/in vivo* rumen fermentation.

Compounds	Nature of experiment (purified MCR or *in vitro* rumen fermentation)	Methane Inhibition	Effect on propionate/H_2_/methanogenic population	References
Nitro compounds				
3-Nitrooxypropanol (3NOP)	*In silico*, *in vitro* and *in vivo* study proves that 3-NOP preferably binds into the active site of MCR.	Up to 30% of methane was decreased in the *in vivo* model	Inhibited the growth of pure methanogenic cultures	Duin et al. (2016)
N-[2-(Nitrooxy)ethyl]-3 pyridinecarboxamide (NPD)	*In vitro* rumen fermentation	Methane was significantly decreased at higher concentrations	Higher concentrations decreased methanogenic archaea, and H_2_ and propionate production were also increased	Jin et al. (2017)
Nitroglycerin	*In vitro* rumen fermentation	No methane was detected at higher concentrations	Methanogens growth was inhibited using high dosage. An increase in propionate and H_2_ concentrations was also recorded	Jin et al. (2017)
Nitrophenol and 5-Nitrobenzimidazol	*In vitro* rumen fermentation	Up to 60% of methane was reduced compared to the control	Propionate production was increased	Kheddouma et al. (2018)
Nitroethane	*In vitro* rumen fermentation	>97%	H_2_ production increased	Zhang et al. (2020)
2-Nitroethanol	*In vitro* rumen fermentation	>97%	H_2_ production increased	Zhang et al. (2020)
2-nitro-1-propanol	*In vitro* rumen fermentation	Up to 59%	H_2_ production increased	Zhang et al. (2020)
Ethyl 2-nitropropionate	*In vitro* rumen fermentation	No methane was detected at higher concentrations	H_2_ and propionate production increased	Ochoa-García et al. (2024)
Ethyl nitroacetate	*In vitro* rumen fermentation	>99%	H_2_ and propionate production increased	Ochoa-García et al. (2024)
2,2-dimethyl-3-(nitrooxy) propanoic (DNP)	*In vitro* rumen fermentation	Methane was not detected at higher concentrations	Both low and high concentrations decreased the methanogenic population. An increase in H_2_ and propionate production was also observed	Jin et al. (2017)
Halogenated compounds				
2-bromoethanesulfonate (BES)	The compound was tested against purified MCR.	Up to 50%		Gunsalus et al. (1978)
Bromopropanesulfonate (BPS)	The compound inhibited the MCRred1 state of purified MCR. Results were concluded with the help of EPR			Rospert et al. (1992)
7-Bromoheptanoylthreonine phosphate	Caused inhibition of the MCRred1 state, which was concluded with EPR spectroscopy			Rospert et al. (1992)
Chloroethanesulfonate	Serving as an analog of CH_3_-S-CoM and targeting MCR from *M. thermoautotrophicum*.	Up to 50% inhibition, but required at higher concentrations compared to BES		Gunsalus et al. (1978)
Sodium 2-(allysulfanyl)ethanesulfonate and Sodium 2-thiocyanatoethanesulfonate	Both compounds inhibited the activity of MCR due to oxidation of Ni, which was analyzed with EPR spectroscopy			Goenrich et al. (2004)
Ammonium 2-(trifluoromethylsulfanyl)ethanesulfonate and Disodium 2-selenolatoethanesulfonate	The activity of MCR was suppressed with both compounds, as evaluated with the help of EPR spectroscopy.			Goenrich et al. (2004)
Natural compounds				
Rosmarinic acid	*In silico* analysis against MCR followed by *in vitro* rumen fermentation	Up to 15%	The relative abundance of the genus *Methanobrevibacter* decreased	Liu et al. (2024)
Bromoform	*In vitro* rumen fermentation	Up to 94%	The methanogenic population decreased	Gyeltshen et al. (2025) and Romero et al. (2023)
Limonene	*In vitro* rumen fermentation	Up to 22%	Propionate concentration decreased	Joch et al. (2015)
α-pinene	*In vitro* rumen fermentation	Up to 28%	Propionate concentration decreased	Joch et al. (2015)
Eugenol	*In vitro* rumen fermentation	Up to 24%	Propionate concentration decreased	Joch et al. (2015)
*p*-cymene	*In vitro* rumen fermentation	Up to 19%	Propionate concentration decreased	Joch et al. (2015)
Citral	*In vitro* rumen fermentation	Up to 43%	Propionate concentration decreased	Joch et al. (2015)
Lovastatin	*In vitro* rumen fermentation	Up to 40%	The methanogenic population decreased	Soliva et al. (2011)
Capric acid	*In vitro* rumen fermentation	Up to 85%	Propionate concentration increased	Goel et al. (2009)

Bromoethanesulfonate (BES) is another competitive compound that has been studied for a long time to decrease methane emissions by targeting MCR (Karnati et al., 2009). The effect of it have been widely studied in *in vitro* models; however, its evaluation in *in vivo* systems is limited due concerns about potential toxic effects. BES mimics the structure of CH_3_-S-CoM as it has the same ethane-sulfonate tail but instead of thiomethyl group it has a Br atom. For BES, The proposed mechanism of action is that the Ni(I) performs a nucleophilic substitution on C-Br leading to the formation of Ni-C bond leaving the Br group as a free radical along with changing the transition state of Ni(I) to oxidized Ni(III). As the Br radical is not the same as methyl radical released by CoM, it cannot accept a free electron from CoB and cannot convert into methane; thus, halting the methane-forming reaction (Gunsalus et al., 1978). Gunsalus et al. (1978) reported that BES can inhibit up to 50% of methane production from a purified MCR. Jeong et al. (2024) reported that BES reduced methane up to 85% in *in vitro* rumen fermentation. In another study, BES inhibited methane production by more than 95% and increased H_2_ production. Order *Methanobacteriales* and *Methanomicrobiales* decreased, but it did not affect the population density of total bacteria (Lee et al., 2009). These studies provide us with a baseline for designing a compound that specifically targets MCR and can inhibit the methane production without affecting the beneficial rumen microbes.

## 8 Natural inhibitors (plant-derived bioactive compounds against MCR)

Many plant-derived compounds have shown excellent diverse potential to reduce CH_4_ production through affecting microbial populations (particularly methanogens). Nowadays, in *silico* studies have increasingly focused on identifying plant-derived compounds with the potential to inhibit MCR function (Saluguti et al., 2025). Rosmarinic acid was used for mitigating methane emissions from ruminants by using *in silico* and *in vitro* approach. Molecular docking was used to identify the inhibitory potential of rosmarinic acid against MCR. *In vitro* supplementation of rosmarinic acid was used, which decreased methane emissions up to 15% and increased propionate production with increasing supplementation levels (Liu et al., 2024). In another *in silico* study, effect of different phytochemicals from 11 different plants were evaluated against MCR. Molecular docking analysis revealed that biotin (−9.38 kcal/mol), *α*-cadinol (−8.16 kcal/mol), and rosmarinic acid (−10.71 kcal/mol) showed the highest binding energy against the MCR protein (Dinakarkumar et al., 2021). Another *in silico* study reported the anti-methanogenic effect of bioactive compounds present in *Moringa olifera* by targeting the MCR protein. Molecular docking analysis revealed that Tetradecanoic acid has the highest binding energy value of −110.36 kj/mol, followed by Niazimisin with −133.98 kJ/mol (Khusro et al., 2020). Anti-methanogenic effect of compounds present in safflower oil was investigated by targeting MCR with the help of *in silico* docking approach. Most compounds showed low binding interaction with the protein (Khusro et al., 2022). Inhibitory potential of compounds present in rhubarb plant was evaluated against MCR protein. A total of 35 compounds from plant were selected to access their binding potential with active site of MCR. Docking results revealed that the highest binding energies were 6.92 kj/mol, −5.26 kcal/mol, and −5.61 kcal/mol with the target protein MCR (Arokiyaraj et al., 2019). No doubt, natural compounds have exhibited excellent inhibitory activity against MCR under *in silico* and *in vitro* conditions (Table 2). However, *in vivo* experiments with larger cohorts of animals are required to optimize the dose and assess long-term effects on microbiome, rumen fermentation shifts, and CH_4_ emissions.

## 9 Challenges

Under normal conditions, methanogenesis acts as a primary hydrogen sink in the rumen. Suppressing methane production with the help of inhibitors can result in hydrogen accumulation inside the rumen, which can cause fermentation imbalance (Ungerfeld, 2020). Excess hydrogen may also inhibit fiber-digesting microbes and affect overall feed efficiency (Schulmand and Valentino, 1976). There is always a risk when using new compounds, as they are new to the field and little is known about their safety (Lileikis et al., 2023). The key concerns related to the use of new compounds besides methane inhibition are (1) how they will affect the meat and milk quality, (2) whether the residues of these compounds in milk will have any adverse effects on human health, or (3) will they carry any carcinogenic or toxic properties with them that will affect the animal over the long term use (Króliczewska et al., 2023). Mass production of compounds that pass rigorous testing is also a difficult task. Some compounds are difficult to synthesize on a large scale, and even when they can be, their cost is so high that a local farmer would hesitate to buy them and add them to an animal’s feed (Manuelian et al., 2023). Delivery of compounds to their designated target is another challenge, as various rumen microorganisms degrade such compounds before they reach MCR, as explained by Duin et al. (2016), that for the *in vivo* model, a large dosage of 3-NOP was required to inhibit methanogenesis compared to its effect on pure methanogenic cultures confirmed by Zhang et al. (2020) that nitro compounds can be cleaved into nitrites by rumen microorganisms. Compounds that have been solely evaluated for their potential effects on *in vitro* rumen fermentation and not tested against purified MCR, their mode of action is still not known despite their potent effects on VFA, methane, and H_2_ production. Commercial status of these tested compounds is still unknown as most of these compounds have been used under *in vitro* trials and their *in vivo* validation is pending before these can be considered for commercial applications.

In most lab trials, compounds have been tested to target methanogenic populations or MCR activity, but they also affect other beneficial rumen microbial species, including *Fibrobacter succinogenes*, *Ruminococcus albus*, and *Ruminococcus flavefaciens* (Martinez-Fernandez et al., 2016). This decreases the complete degradation of feed into a useful energy source, as indicated by a decrease in VFAs production. Understanding and mitigating these broader microbiome effects are essential to ensure that methane reduction strategies do not compromise animal productivity or health (Martin and Macy, 1985).

## 10 Opportunities

Despite the risks of hydrogen buildup and decreased fermentation quality when using synthetic compounds, redirecting hydrogen away from methanogenesis into other beneficial metabolic sinks provides a key opportunity to improve animal productivity and minimize methane emissions (Wang et al., 2018). *Selenomonas ruminantium* uses the succinate pathway to convert fumarate to succinate and then to propionate, consuming reducing equivalents (H₂) (Paynter and Elsden, 1970). *Veillonella parvula* also ferments lactate and uses hydrogen to form propionate (Ng and Hamilton, 1971). *Megasphaera elsdenii* converts lactate to propionate via the acrylate pathway, which utilizes reducing power from H₂ (Hino et al., 1994). As mentioned in Table 2, inhibition of methanogenesis is accompanied by an increase in propionate production, as propionate formation serves as a major metabolic sink for H₂ in the rumen. In a study, Jeong et al. (2024) increased H_2_ production by inhibiting methane with BES and converted it into propionate using *Lactiplantibacillus plantarum*, *Megasphaera elsdenii*, *Selenomonas ruminantium*, and *Acidipropionibacterium thoenii*. Although facing regulatory hurdles, compounds such as 3-NOP have shown promising results in the livestock sector with no harmful residues detected in milk or meat at tested concentrations (Zhang et al., 2018; Króliczewska et al., 2023). Once approved, compounds with proven safety margins will offer a dependable solution for enhancing animals’ productivity, helping convince farmers to use such compounds as feed additives (Wanapat et al., 2013). Compounds that affect only methanogens without affecting the other rumen microbiota should be allowed for further evaluation. One of the best examples is 3-NOP, which affects only MCR activity without affecting the other microorganisms found in the rumen (Duin et al., 2016). Degradation-related issues can be minimized by encapsulating the inhibitory compounds within nanoparticles (Munin and Edwards-Lévy, 2011). Amin et al. (2021) proved that microencapsulated cinnamaldehyde essential oil degrades at a lower rate compared to free essential oil in *in vitro* rumen fermentation. Heo et al. demonstrated that the stability of conjugated linoleic acid can be improved through microencapsulation; it cannot be degraded by *Butyrivibrio fibrisolven* and is readily available to animals, thereby improving their health. This reveals that encapsulation approaches can be very effective to protect compounds from premature degradation while enhancing their stability at rumen pH. Nowadays, *in silico* studies offer a valuable and cost-effective approach to address biological problems, allowing researchers to virtually screen and evaluate a large number of compounds before *in vitro* or *in vivo* testing. For instance, molecular docking and simulation studies are increasingly being used to investigate the interactions of various phytochemicals with MCR as mentioned above, providing insights into their potential to inhibit methanogenesis (Dinakarkumar et al., 2021). Latest developments in Omics techniques, particularly genomics, metagenomics, and metabolomics, offer unique opportunities to explore the structure–activity relationship of MCR, biochemical pathways involved, and diversity of methanogens. Genome editing, such as CRISPR-Cas9, can be employed for bioengineering of the microbiome. To date, CRISPR-Cas9 systems have been optimized for bacteria/eukaryotes, but delivery and expression in archaeal systems (especially rumen methanogens) are limited. Some archaea even lack compatible promoters and repair systems for Cas9. Recently, CRISPR-Cas systems derived from archaea themselves (e.g., *Cas9 from Haloarcula hispanica*, *Cpf1 from Sulfolobus*) are being optimized for future applications, which can pave the way for technological interventions to manipulate the MCR enzyme or associated proteins to reduce/block the methanogenesis pathway. No doubt, direct MCR editing in rumen methanogens is not yet feasible, current strategies to reduce methane emissions include: feeding interventions (e.g., 3-NOP, seaweed algae (Asparagopsis), nitrates), breeding for low-methane animals using genomic selection, Vaccines targeting methanogens or MCR enzyme epitopes, and CRISPR editing of host-associated bacteria to modify rumen fermentation toward propionate instead of methane.

## 11 Conclusion

Methane emissions from the livestock sector pose a serious threat to global climate stability, and mitigating them is an urgent need of today. Feed additives, including tannins, saponins, or PUFAs, have shown potential to reduce enteric methane emissions; however, they do not offer a permanent solution as their continuous use alters rumen communities. The MCR remains the main target for inhibiting methane emissions as it catalyzes the final CH_4_-forming step in methanogens and has shown promising results with few inhibitors tested so far. By targeting MCR, the CH_4_ emanation issue in ruminants can be solved without affecting the overall rumen microbiota. Following the success of 3-NOP and BES, questions remain about their long-term safety, which warrants further studies. Current research focuses on plant-derived compounds, using *in silico* approaches. Their ADMET (Absorption, Distribution, Metabolism, Excretion, and Toxicity) properties can be predicted using web-based servers, so they can be evaluated in *in vitro* and *in vivo* models, which seems the most promising approach. In the next decade, the use of OMICS will drive anti-methanogenic strategies, as genome-editing techniques such as CRISPR-Cas9 are also being optimized for the archaeal system and can be employed in the future to determine their potential to knock out or modify MCR or associated proteins to inhibit methanogenesis. Additionally, CRISPR-Cas12a systems sometimes perform better in archaea due to simpler PAM requirements, whereas homologous recombination with selection markers remains the standard for methanogen editing. Developing methanogen-specific vaccines is also a promising strategy to induce a host immune response, which secretes specific antibodies in the rumen through saliva, directly inhibiting the activity of methanogenic archaea or interrupting their hydrogenotrophic activity. Collectively, these strategies will further shape technological interventions to reduce methane emissions from ruminants, enhance feed efficiency, and mitigate environmental impacts.
